# The *Gauche* Effect in XCH_2_CH_2_X Revisited

**DOI:** 10.1002/cphc.202100090

**Published:** 2021-02-25

**Authors:** Daniela Rodrigues Silva, Lucas de Azevedo Santos, Trevor A. Hamlin, Célia Fonseca Guerra, Matheus P. Freitas, F. Matthias Bickelhaupt

**Affiliations:** ^1^ Department of Theoretical Chemistry Amsterdam Institute of Molecular and Life Sciences (AIMMS) Amsterdam Center for Multiscale Modeling (ACMM) Vrije Universiteit Amsterdam De Boelelaan 1083 1081 HV Amsterdam (The Netherlands; ^2^ Departamento de Química Instituto de Ciências Naturais Universidade Federal de Lavras 37200-900 Lavras-MG Brazil; ^3^ Leiden Institute of Chemistry Gorlaeus Laboratories Leiden University Einsteinweg 55 2333 CC Leiden (The Netherlands; ^4^ Institute for Molecules and Materials (IMM) Radboud University Heyendaalseweg 135 6525 AJ Nijmegen (The Netherlands

**Keywords:** activation strain model, bond theory, conformational analysis, energy decomposition analysis, gauche effect

## Abstract

We have quantum chemically investigated the rotational isomerism of 1,2‐dihaloethanes XCH_2_CH_2_X (X = F, Cl, Br, I) at ZORA‐BP86‐D3(BJ)/QZ4P. Our Kohn‐Sham molecular orbital (KS‐MO) analyses reveal that hyperconjugative orbital interactions favor the *gauche* conformation in all cases (X = F−I), not only for X = F as in the current model of this so‐called *gauche* effect. We show that, instead, it is the interplay of hyperconjugation with Pauli repulsion between lone‐pair‐type orbitals on the halogen substituents that constitutes the causal mechanism for the *gauche* effect. Thus, only in the case of the relatively small fluorine atoms, steric Pauli repulsion is too weak to overrule the *gauche* preference of the hyperconjugative orbital interactions. For the larger halogens, X⋅⋅⋅X steric Pauli repulsion becomes sufficiently destabilizing to shift the energetic preference from *gauche* to *anti*, despite the opposite preference of hyperconjugation.

## Graphical abstract

**Beyond hyperconjugation**! State‐of‐the‐art quantum chemical analyses reveal that, in 1,2‐dihaloethanes, hyperconjugation always favors the *gauche* conformation. It is the steric (Pauli) repulsion that is behind the trend known as the *gauche* effect: weak repulsion (X = F) leaves the *gauche* preference intact, whereas stronger repulsion (X = Cl, Br, I) shifts the preference to *anti*.

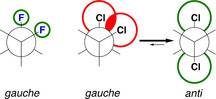

## 1 Introduction

**Figure 1:**
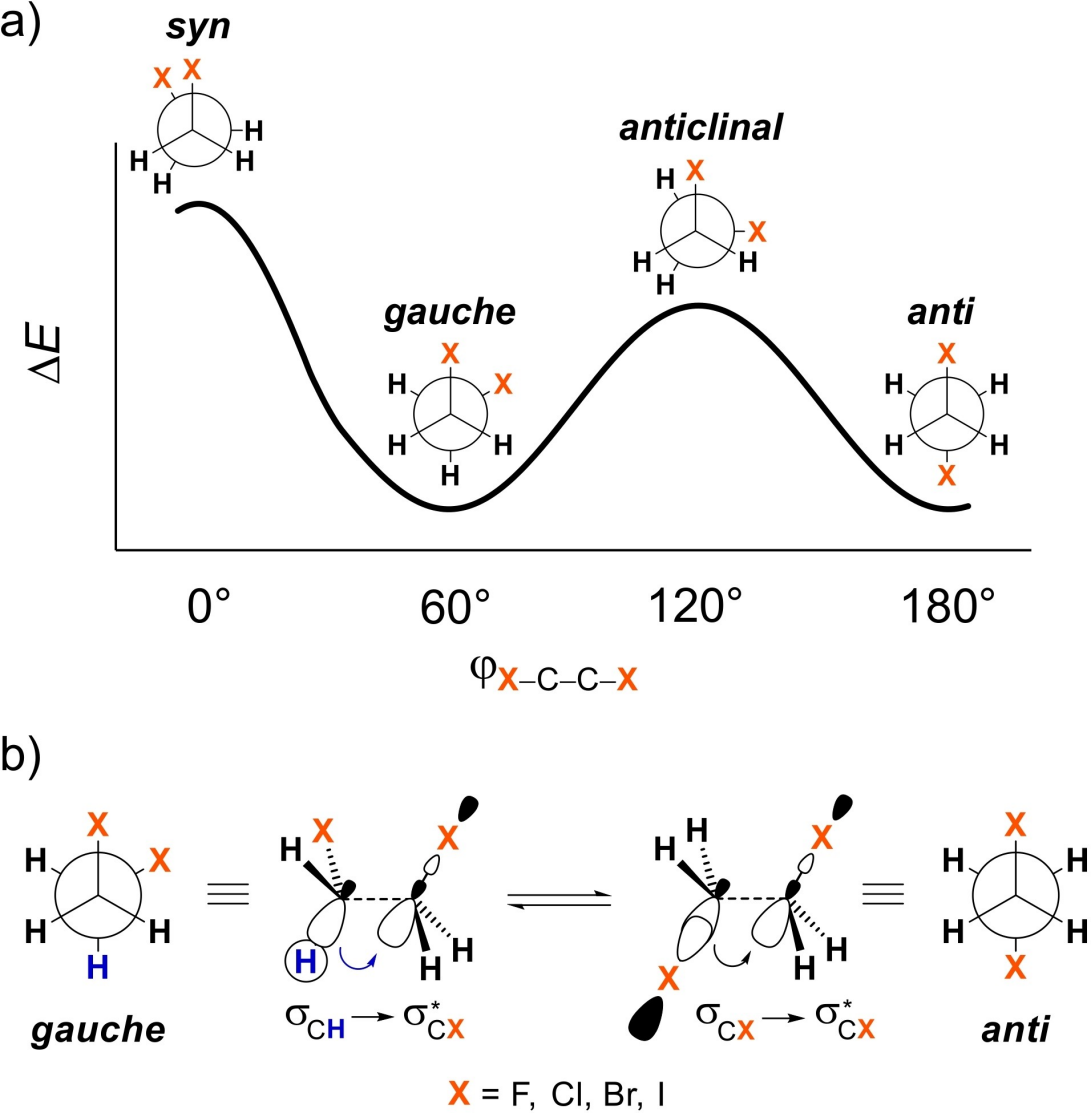
a) Stationary points in the energy profile for rotation around the C−C bond of 1,2‐dihaloethanes and b) main orbital interactions affecting the *gauche*‐*anti* equilibrium.

The energy profile for rotation around the C−C bond in 1,2‐disubstituted ethanes features four stationary points, that is, two staggered conformers (*gauche* and *anti*) connected via two eclipsed transition states (*syn* and *anticlinal*), as schematically illustrated in Figure 1a.^[1]^ Depending on the nature of the substituted groups, the equilibrium can shift to favor either the *gauche* or the *anti*‐conformer.^[2]^ The so‐called *gauche* effect, a term coined by Wolfe in 1972,^[3]^ is the phenomenon that the *gauche* conformer is energetically more favorable than the *anti*‐conformer in cases where X is an electron‐withdrawing group (usually containing atoms from the second period of the periodic table, such as nitrogen, oxygen, or fluorine).^[4]^ The *gauche* effect has been observed in a variety of molecules (especially those containing an organic fluorine)^[5]^ and has been used as a tool for controlling the conformational preference in the design of organic compounds towards specific molecular properties.^[6]^

In the currently accepted model, the *gauche* effect arises from stabilizing hyperconjugative^[7]^ interactions between antiperiplanar σ orbitals (see Figure 1b).^[8]^ This picture was developed to explain experimental evidences on the conformational behavior of the 1,2‐difluoroethane,^[9]^ the simplest molecule that exhibits the *gauche* preference.^[10]^ In this hyperconjugation model, the higher stability of the *gauche* conformer is attributed to the antiperiplanar charge‐transfer from the filled σ_C−H_ orbital to the empty σ^*^_C−F_ orbital, which is stronger than the corresponding charge‐transfer from the filled σ_C−F_ to the empty σ^*^_C−F_ in the *anti*‐conformer.^[8]^ For heavier halogens, it is argued that this trend should be reversed, because of the better electron‐donor and electron‐acceptor capabilities of σ_C−X_ and σ^*^_C−X_ orbitals as X goes from F to Cl, Br, or I.^[11]^ However, recent reports have shed more light on the role of other forces behind this effect. Baranac‐Stojanović^[12]^ pointed out that stabilization of the *gauche* conformer is caused by orbital and also electrostatic interactions, Thacker and Popelier^[13]^ attributed it solely to electrostatics, while Martín‐Pendás and coworkers^[14]^ explained it based on both electrostatic and exchange‐correlation interactions.^[15]^

In view of this ongoing and highly relevant controversy, we have investigated the origin of the *gauche* effect within the framework of quantitative Kohn‐Sham molecular orbital (KS‐MO) theory using the series of 1,2‐dihaloethanes XH_2_C−CH_2_X (X = F, Cl, Br, I, see Figure 1). Herein, we show that, at variance to the currently prevailing model, the switch in preference from *gauche* for X = F to *anti* for X = Cl−I, is caused by the increasing steric demand of the substituent X, as the latter descends down a group in the periodic table. This trend does not originate from hyperconjugation, which we show to always favor *gauche* but only overrule steric (Pauli) X⋅⋅⋅X repulsion in the *gauche* conformation for the smallest substituent X, in our model systems, the second‐row fluorine atom. We also highlight how geometrical relaxation, in particular, the effect of the variation of the C−C bond length, upon internal rotation around this bond can mask the change in the various orbital and electrostatic interactions and needs to be taken into consideration to properly identify causalities.

## 2 Results and Discussion

**Figure 2:**
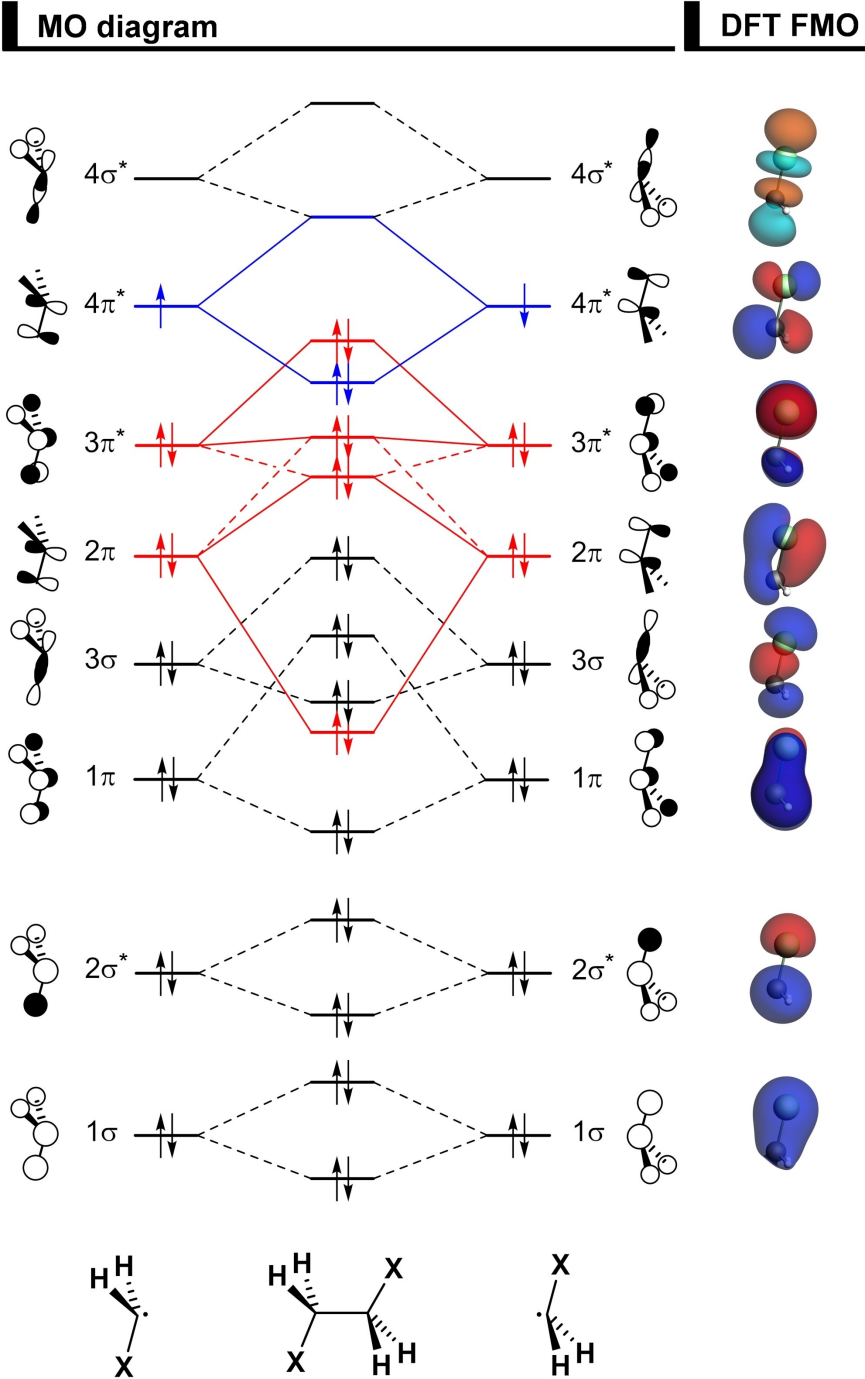
MO diagram for the formation of the 1,2‐dihaloethanes XH_2_C−CH_2_X (X = F, Cl, Br, I) from two open‐shell CH_2_X^.^ fragments, along with the fragment molecular orbitals (FMO) depicted as quantitative 3D plots (isovalue = 0.04) for CH_2_Cl^.^, computed at ZORA‐BP86‐D3(BJ)/QZ4P.

To understand how the XH_2_C−CH_2_X bonding mechanism determines conformational preferences, we have analyzed this bond explicitly for all four 1,2‐dihaloethanes in terms of two open‐shell CH_2_X^.^ fragments forming a C−C electron‐pair bond in various conformations. The MO diagram with the valence orbitals of the CH_2_X^.^ fragments is provided in Figure 2 (see Figure S1 for more details of CH_2_X^.^ and the better known CH_3_X molecular orbitals). The overall bond energy Δ*E* has been divided into two major components using the activation strain model (ASM):^[16]^ the strain (Δ*E*_strain_) that results from the distortion of the two CH_2_X^.^ radicals from their equilibrium structure to the geometry they acquire in the XH_2_C−CH_2_X molecule and the actual interaction (Δ*E*_int_) between the deformed radical fragments. The interaction energy Δ*E*_int_ was further decomposed using our canonical energy decomposition analysis for open‐shell fragments (EDA)^[17]^ into four energy terms that can be associated with the following physical factors: classical electrostatic interaction (Δ*V*_elstat_), Pauli repulsive orbital interactions (Δ*E*_Pauli_) between closed‐shell orbitals which is responsible for steric repulsion, stabilizing orbital attractions (Δ*E*_oi_) that account, among others, for electron‐pair bonding as well as donor‐acceptor interactions, and corrections for dispersion interactions (Δ*E*_disp_). For the purpose of clarity, the above‐mentioned energy terms along the internal rotation around the C−C bond are considered relative to the *syn* conformation (*i. e*., represented as a ΔΔ*E*), since the latter represents the global energy maximum conformation in all cases. All calculations were performed using ZORA‐BP86‐D3(BJ)/QZ4P^[18]^ as implemented in the Amsterdam Density Functional (ADF) program,^[19]^ and PyFrag 2019 to facilitate all ASM and EDA analyses.^[20]^

**Figure 3:**
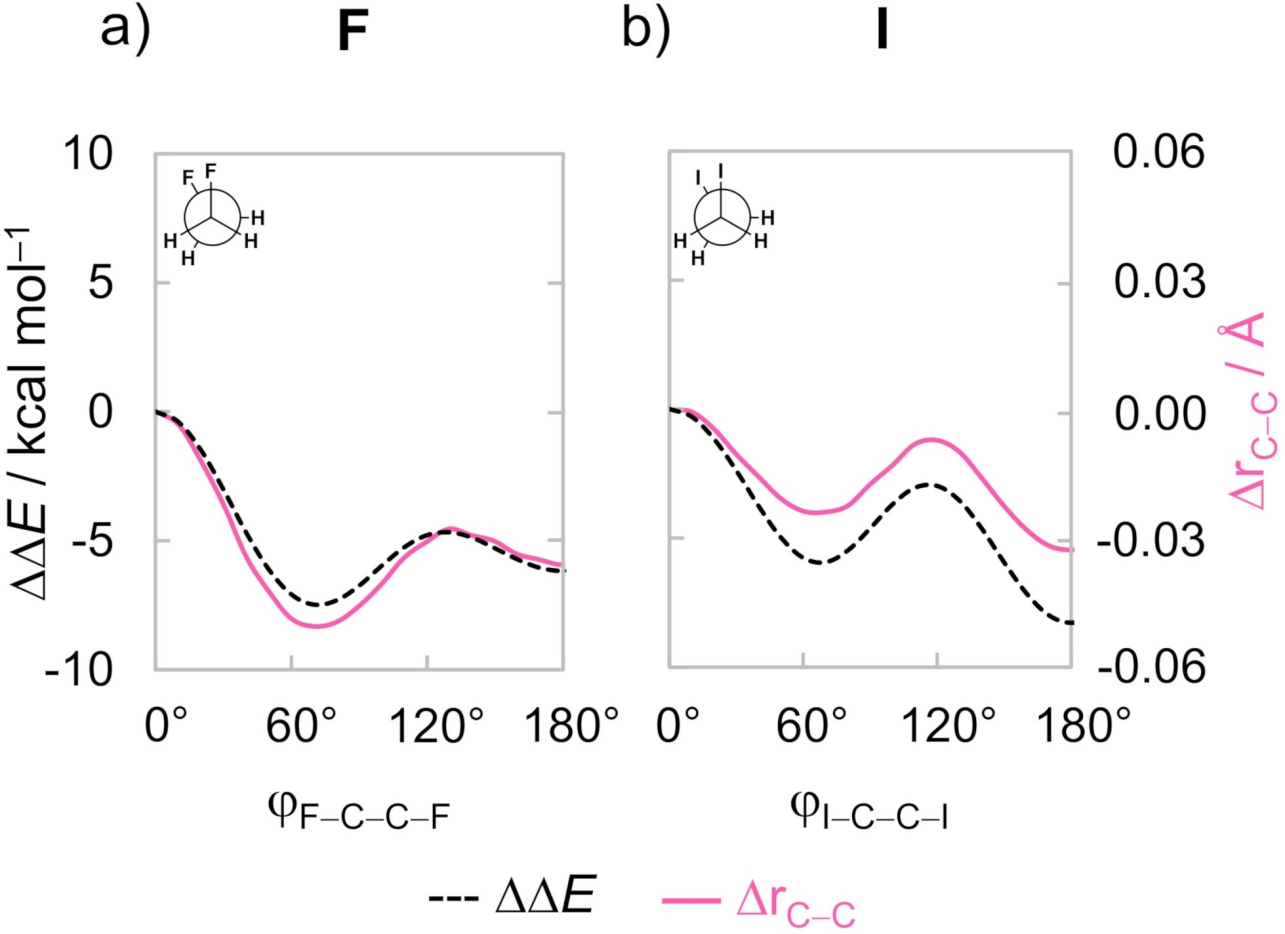
Rotational energy profile and C−C bond length variation as a function of the ϕ_X−C−C−X_ dihedral angle of the 1,2‐difluoro and 1,2‐diiodoethanes. Computed at ZORA‐BP86‐D3(BJ)/QZ4P.

Our rotational energy profiles of 1,2‐difluoro and 1,2‐diiodoethanes are given in Figure 3 (full data on all model systems can be found in Figure S2 in the Supporting Information). Firstly, we note the well‐known energy profile of 1,2‐dihaloethanes XH_2_C−CH_2_X,^[11]^ that is, for 1,2‐difluoroethane (X = F), the *gauche* conformer is the global energy minimum, whereas the *anti*‐conformer is the global energy minimum for all heavier 1,2‐dihaloethanes (X = Cl, Br, I). The torsion angle ϕ_X−C−C−X_ (where X = F, Cl, Br, I) of the *gauche* conformer is shifted from 60° to *ca*. 70°, in good agreement with the experimental value of 71.0(3)° for 1,2‐difluoroethane (X = F).^[21]^ The C−C bond length directly correlates with conformational stabilities and is shorter in the energy minima and longer in the energy maxima (see Figure 3). For example, in 1,2‐difluoroethane (X = F), the C−C bond shortens from *syn* (1.556 Å) to *gauche* (1.506 Å), then lengthens going towards the *anticlinal* conformation (1.526 Å) and shortens again at the *anti*‐conformer (1.520 Å). Similar, though less pronounced effects are observed for the heavier halogens.

Thus, the variation in C−C bond length along internal rotations is significant (*e. g*., for 1,2‐difluoroethane, it spans 0.05 Å). In the following, we address the question of where this breathing in C−C bond distance between short in minima and long in maxima comes from, and how it affects the individual interaction mechanisms and their EDA energy terms.

### 2.1 Geometrical Relaxation and Rotational Energy Profile

Our analyses reveal an interesting interplay of effects in which steric Pauli repulsion is the dominant term behind both the raise in energy and the expansion of the C−C bond at eclipsed conformations. The results of these analyses also highlight the importance of separating changes in bonding mechanism, and thus in EDA terms, associated with the internal rotation from further changes in bonding, and thus in EDA terms, due to the further geometrical relaxation (in particular, C−C bond‐length variation) that occurs in response to the internal rotation, because this further geometrical relaxation again modifies and thus hides the original causal factors.

**Figure 4:**
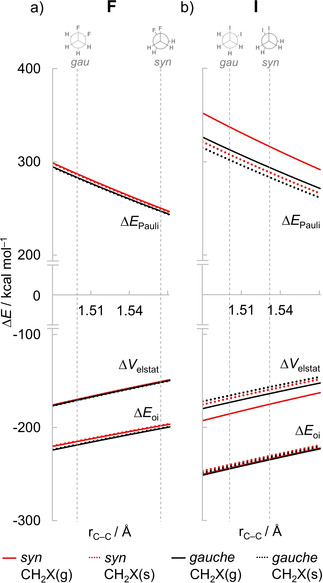
EDA of the interaction between two open‐shell CH_2_X^.^ fragments in 1,2‐difluoro and 1,2‐diiodoethanes as a function of the C−C separation, computed at ZORA‐BP86‐D3(BJ)/QZ4P. Vertical dashed lines denote the equilibrium bond distances of the *syn* and *gauche* conformations.

Figure 4 shows how the different energy components (*i. e*., Pauli repulsion, electrostatics and orbital interactions) vary as a function of C−C separation for the *syn* and *gauche* conformations of 1,2‐difluoro and 1,2‐diiodoethanes (full data on all model systems can be found in Figure S3 in the Supporting Information). Note that, in a fully relaxed rotation around the C−C bond, not only the C−C bond length changes, but also the geometry of the CH_2_X^.^ fragments which becomes increasingly pyramidalized in the eclipsed conformation to mitigate the buildup of steric Pauli repulsion (*vide infra*). Therefore, to separate the effect of C−C bond length variation from the effect of CH_2_X^.^ bending, we first focus on the curves with fixed CH_2_X^.^ geometry as in the *gauche* conformer CH_2_X(g) (solid lines, red for *syn* and black for *gauche*). Note that the upward slope of the Δ*E*_Pauli_ curve is larger than the downward slopes of the Δ*V*_elstat_ and Δ*E*_oi_ curves in all cases, which means that Δ*E*_Pauli_ changes faster as a function of the C−C distance. Also, the Pauli repulsion is strongest in the *syn* conformation for all distances shown and the gradient of the red line is larger than of the black line, that is, the increase of Δ*E*_Pauli_ with the shortening of the C−C bond length is greater in the *syn* case (see Figure S4 in the Supporting Information for the derivative of the different energy terms with respect to the C−C bond length). The larger gradient of the Δ*E*_Pauli_ curve in the *syn* conformation will drive the molecule towards a longer equilibrium C−C bond length.^[22]^ Therefore, steric Pauli repulsion is the reason why the C−C bond is longer at the *syn* conformation. The picture remains essentially the same if we consider the effect of CH_2_X^.^ bending to the *syn* geometry CH_2_X(s) (dotted lines, red for *syn* and black for *gauche*). The magnitude of all energy terms is however smaller since, in its *syn* geometry, CH_2_X^.^ is more bent away from the C−C bond region which reduces the Pauli repulsion (but also other interactions) between the fragments.

**Figure 5:**
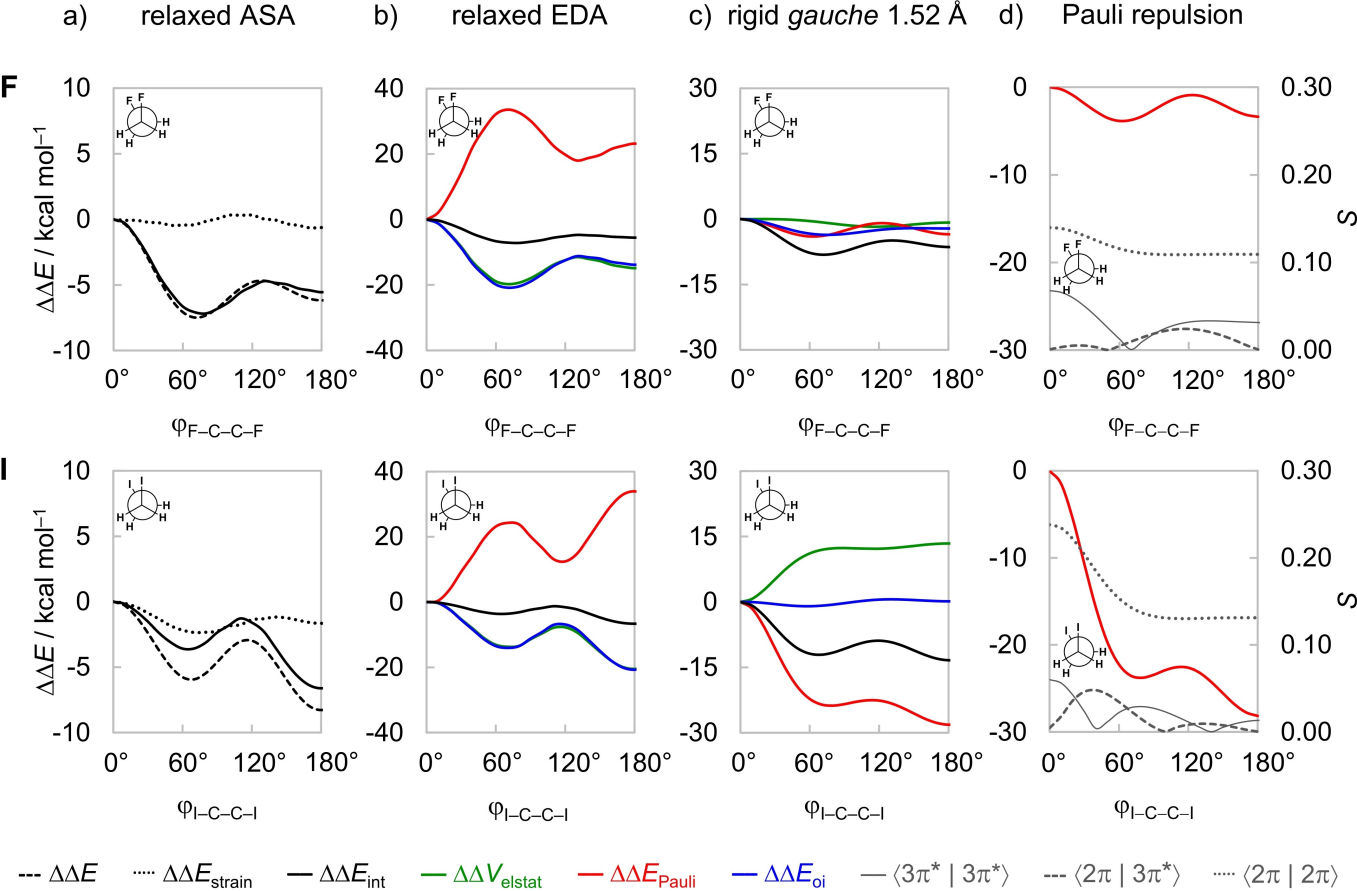
Activation strain (ASA) and energy decomposition analyses (EDA) as a function of the ϕ_X−C−C−X_ dihedral angle of the 1,2‐difluoro and 1,2‐diiodoethanes. a) ASA and b) EDA for fully relaxed rotation, c) EDA and d) key closed‐shell‐closed‐shell overlaps for rigid rotation with CH_2_X^.^ fragments in *gauche* geometry but with C−C distance set to 1.52 Å. Computed at ZORA‐BP86‐D3(BJ)/QZ4P.

**Figure 6:**
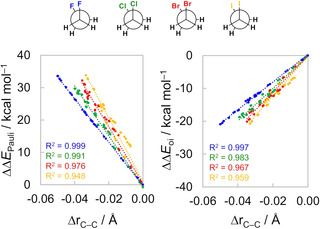
Correlation between the C−C bond stretching with steric Pauli repulsion and orbital interactions during rotation around the C−C bond. Computed at ZORA‐BP86‐D3(BJ)/QZ4P.

Next, we analyze how geometrical relaxation, in particular, the change in C−C bond length, affects the interpretation of the energy profile for internal rotation around this bond of the 1,2‐dihaloethanes. Our results reveal that all energy components are strengthened in the global energy minima because of the shorter C−C bond in these conformations, that is, the observed trends in ΔΔ*V*_elstat_, ΔΔ*E*_Pauli_, and ΔΔ*E*_oi_ are simply a function of the C−C distance. Thus, care should be exercised when analyzing relaxed rotational barriers (*i. e*., when all geometrical parameters are flexible to optimize during rotation). The key to understand the trends in the interacting terms is to first take a closer look at the fully relaxed rotation around the C−C bond. As can be seen from Figure 5a for the 1,2‐difluoro and 1,2‐diiodoethanes (full data on all model systems can be found in Figure S5 in the Supporting Information), the ΔΔ*E* originates primarily from a more stabilizing ΔΔ*E*_int_ that favors the *gauche* conformer for X = F and the *anti*‐conformer for X = Cl, Br, I. The ΔΔ*E*_strain_, stemming from the bending of the CH_2_X^.^ fragments, is much smaller and more uniform along the rotation of the C−C bond. Since the interaction energy plays such a critical role in the observed trends, the different contributors to the interaction energy were analyzed in more detail using our EDA.^[17]^ Figure 5b shows that, in all cases, the trends in the interaction energy are equally dictated by the stabilizing orbital and electrostatic interactions (the ΔΔ*E*_oi_ and ΔΔ*V*_elstat_ curves nearly coincide in all points of the rotation around the C−C bond). On first glance, these findings are, indeed, in line with a previous report that attributed the *gauche* effect to both orbital and electrostatic stabilization.^[12]^ However, owing in particular to the C−C bond lengthening (which is caused by a *higher* steric Pauli repulsion at any given C−C distance, *vide supra*), the ΔΔ*E*_Pauli_ term is lowest along this curve in the *syn* conformation. This observation is counterintuitive, as already shown in Figure 4 and stressed in the analysis of the rotation barrier of ethane,^[22]^ in that the steric repulsion is least destabilizing in the eclipsed conformation where the two C−X bonds are pointing in the same direction and the halogen lone‐pairs on each fragment are in closest proximity (see 2π and 3π* orbitals in Figure 2). The trends observed in Figure 5b are dominated by consequence of the C−C bond shortening and stretching, that is, all energy terms are maximized when the C−C bond is shorter (*i. e*., in the staggered conformers, ΔΔ*E*_oi_ and ΔΔ*V*_elstat_ are more stabilizing, and ΔΔ*E*_Pauli_ is more destabilizing) whereas the opposite occurs when the C−C bond is longer (*i. e*., in eclipsed conformers; see Figures 3 and 5b).^[23]^ Figure 6 confirms that the changes in the C−C bond length (Δr_C−C_) correlate with both ΔΔ*E*_Pauli_ and ΔΔ*E*_oi_ terms.

Therefore, to obtain an unbiased picture, it is necessary to perform a numerical experiment in which the strong effect of C−C bond length variation on the energy terms has been eliminated. This can be achieved by performing a rigid rotation around the C−C bond while all geometry parameters but the torsion angle ϕ_X−C−C−X_ are kept unchanged. To this end, let us take the *gauche* conformer of each 1,2‐dihaloethane at its optimum geometry and rotate it from the *syn* to the *anti*‐conformation. To compare all molecules on a more equal footing, we rotate all 1,2‐dihaloethanes from the same C−C bond distance set to 1.52 Å (as in the staggered geometry with the longest C−C bond length, *i. e*., the *anti*‐conformer of the 1,2‐difluoroethane; see Figure 5c). Note that the conformational preferences in the ΔΔ*E*_int_ curve shown in Figure 5c remain the same as in the fully relaxed rotational profiles, that is, ΔΔ*E*_int_ favors *gauche* for X = F and *anti* for X = Cl, Br, and I. The ΔΔ*E*_Pauli_ is a minimum at the staggered conformations and goes to a maximum at the eclipsed conformations, as would be expected. Note that the other energy components have a smaller contribution to the trends in ΔΔ*E*_int_. The ΔΔ*V*_elstat_ term is more stabilizing at the eclipsed conformations (see ref.^[22]^ for a detailed discussion on the behavior of ΔΔ*V*_elstat_ in rotation barriers), and the ΔΔ*E*_oi_ only slightly changes upon rotation around the C−C bond. The same overall trend is found if we rotate all 1,2‐dihaloethanes from their optimum *gauche* or *anti* (Figure S6) as well as *syn* geometries (Figure S7). Note that the strain energy Δ*E*_strain_ vanishes in this analysis because it is constant for geometrically frozen fragments; therefore, ΔΔ*E*_strain_ is zero and ΔΔ*E*_int_ = ΔΔ*E*.

**Figure 7:**
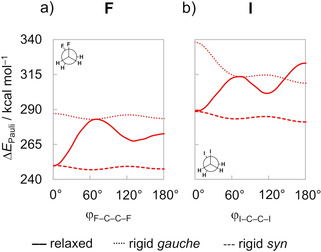
Pauli repulsion as a function of the ϕ_X−C−C−X_ dihedral angle of the 1,2‐difluoro and 1,2‐diiodoethanes. Computed at ZORA‐BP86‐D3(BJ)/QZ4P.

The smallest ΔΔ*E*_Pauli_ for *syn* observed in the flexible rotation (Figure 5b) is, therefore, a result of the elongation of the C−C bond and the bending of the CH_2_X^.^ fragments, that reduces the overlap between the orbitals of the vicinal C−X bonds. Note, for example, that the rigid rotation from the equilibrium *gauche* conformer of the 1,2‐difluoroethane to the *syn* orientation leads to 4.0 kcal mol^−1^ more Pauli repulsion (Figure 7). Then, when we keep the molecule in the *syn* conformation but relax the other geometry parameters, the Pauli repulsion lowers almost 40 kcal mol^−1^, much more than the initial rise of 4.0 kcal mol^−1^. Similar effects can be observed for other 1,2‐dihaloethanes (see Figure S7 for the connection of each EDA term between a staggered and an eclipsed rigid rotation of all 1,2‐dihaloethanes). This highlights that analyses over relaxed rotational barriers, or final equilibrium geometries, only reflect the consequences but not the reason that leads to a given structural preference in the first place. The importance of taking into account the strong effect of geometrical relaxation in the course of a chemical phenomenon when elucidating the physical factors underlying it has already been nicely pointed out in the literature.[^22^, ^24^] Therefore, detailed analyses of the rotational profiles of 1,2‐dihaloethanes performed herein clearly demonstrate that ΔΔ*E*_Pauli_ is the dominant, causal term that determines the observed trends in the variation of the C−C bond length and in the overall rotational energy profile.^[25]^

### 2.2 Steric Pauli Repulsion and the *Gauche* Effect

In the following, we discuss how the above analysis of the rotational energy profiles affects the interpretation of the *gauche* effect observed in the 1,2‐difluoroethane. We show that the fluorine atoms in FCH_2_CH_2_F are too small to cause significant steric Pauli repulsion in the *gauche* conformation and, for this reason, hyperconjugation dominates for X = F.

**Figure 8:**
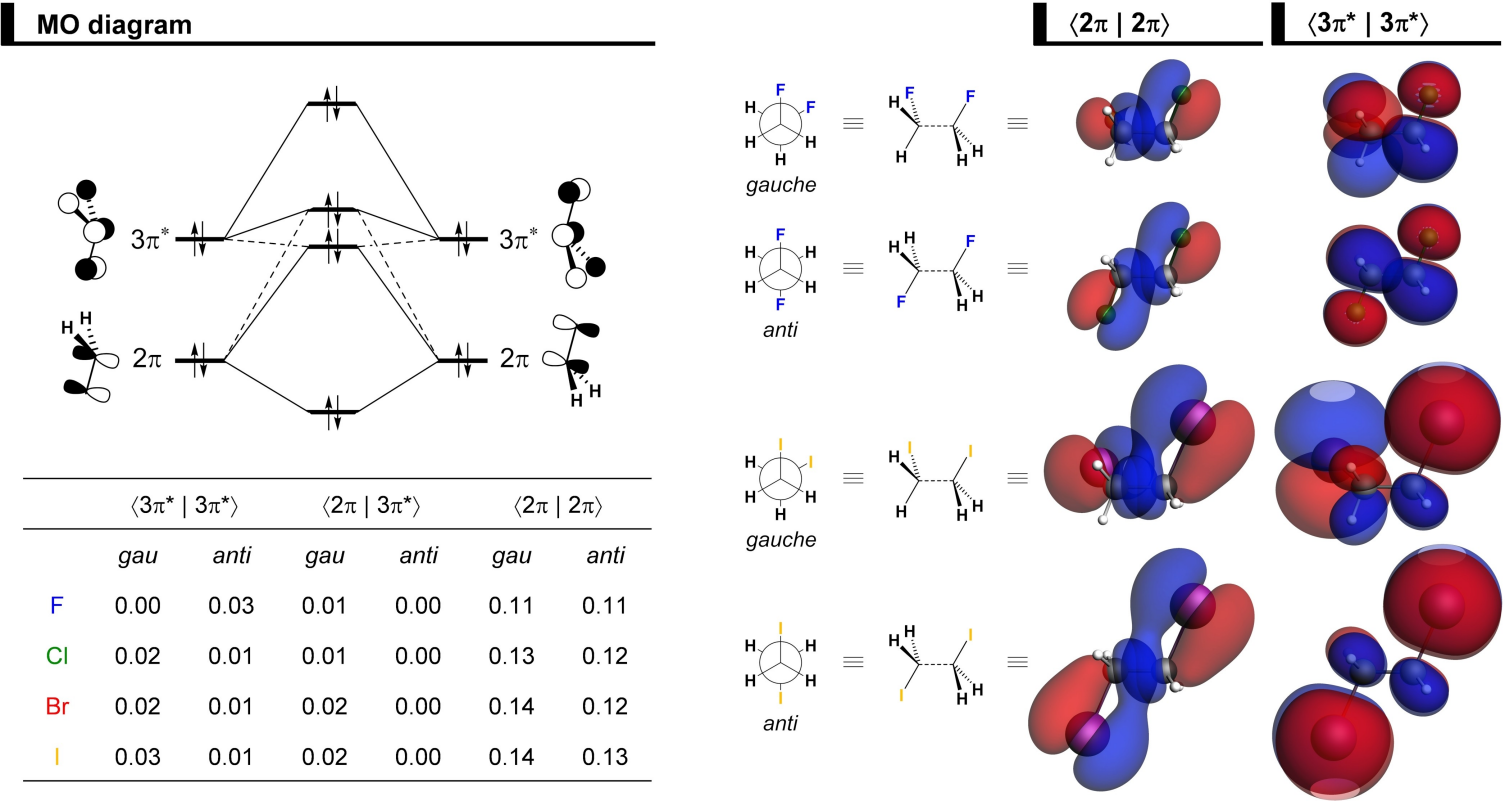
MO diagram and the most significant occupied‐occupied orbital overlaps between the CH_2_X^.^ fragments (where X = F, Cl, Br, I), along with ⟨2π|2π⟩ (isovalue = 0.04) and ⟨3π*|3π*⟩ (isovalue = 0.02) in the *gauche* and *anti*‐conformers of the 1,2‐difluoro and 1,2‐diiodoethane. Analysis in rigid rotation in *gauche* geometry but with C−C distance set to 1.52 Å, computed at ZORA‐BP86‐D3(BJ)/QZ4P.

The most significant closed‐shell−closed‐shell overlaps between the two CH_2_X^.^ fragments contributing to the trend in ΔΔ*E*_Pauli_ (shown in Figure 5c) arise between the well‐known C−X π‐bonding and π*‐antibonding CH_2_X^.^ FMOs,^[26]^ 2π and 3π*, respectively, each of which possesses both, X lone‐pair character as well as carbon 2p amplitude (see Figure 8). ΔΔ*E*_Pauli_ is a maximum at the *syn* because of a larger ⟨2π|2π⟩ overlap, which decreases as the C−C bond is rotated to the *anti*‐conformer (see Figure 5d). This effect is more pronounced for heavier halogens because of the larger spatial extension of their valence n*p* atomic orbitals. Note that the difference in ⟨2π|2π⟩ between *gauche* and *anti*‐conformers also increases as X goes from F to I. For example, for X = F, the ⟨2π|2π⟩ is the same in the *gauche* and *anti*, whereas, for X = Cl, Br, and I, it is larger in the *gauche* (see Figure 8). A similar behavior is found for the ⟨2π|3π*⟩ overlap integral. Thus, the trend in Pauli repulsion determines that the conformational equilibrium gradually shifts to the *anti*‐conformer as the halogen increases in size.^[11]^ Interestingly, only for 1,2‐difluoroethane, the ΔΔ*E*_Pauli_ is slightly larger for *anti* than *gauche* (see Table S1). This can be ascribed to the ⟨3π*|3π*⟩ overlap, in which the amplitude of the 3π* orbital is larger on the less electronegative carbon atom because of the out‐of‐phase mixing of C 2p and F 2p orbitals, resulting in a larger ⟨3π*|3π*⟩ for *anti* than *gauche* (see Figure 8). As the atomic p orbital of the halogen atom goes up in energy (and becomes more diffuse) going down group 17 in the periodic table, the amplitude of the 3π* orbital increases on the halogen atom and, thus, the ⟨3π*|3π*⟩ overlap is smaller for *anti* than *gauche* for X = Cl, Br, and I. At variance to the heavier and effectively larger halogens, the fluorine orbitals are too compact to cause significant overlap and steric Pauli repulsion and, therefore, cannot shift the conformational equilibrium to *anti*.

The important role played by steric Pauli repulsion is not surprising for heavier 1,2‐dihaloethanes (X = Cl, Br, I),^[11]^ but it constitutes an unprecedented physical factor behind the *gauche* effect in 1,2‐difluoroethane (X = F).^[27]^ In contrast to the widespread belief that the fluorine atoms would repel each other in the *gauche* conformation, our analysis at the consistent geometries (*i. e*., all conformations at the same C−C bond length of 1.52 Å) shows that ΔΔ*E*_Pauli_ slightly favors (by 0.4 kcal mol^−1^) the *gauche* over the *anti*‐conformer for X = F (Figure 5d). In this way, any subtle attractive interaction can effectively determine its conformational preference, which is the case of the hyperconjugative interactions used to explain the *gauche* effect in the 1,2‐difluoroethane.

**Figure 9:**
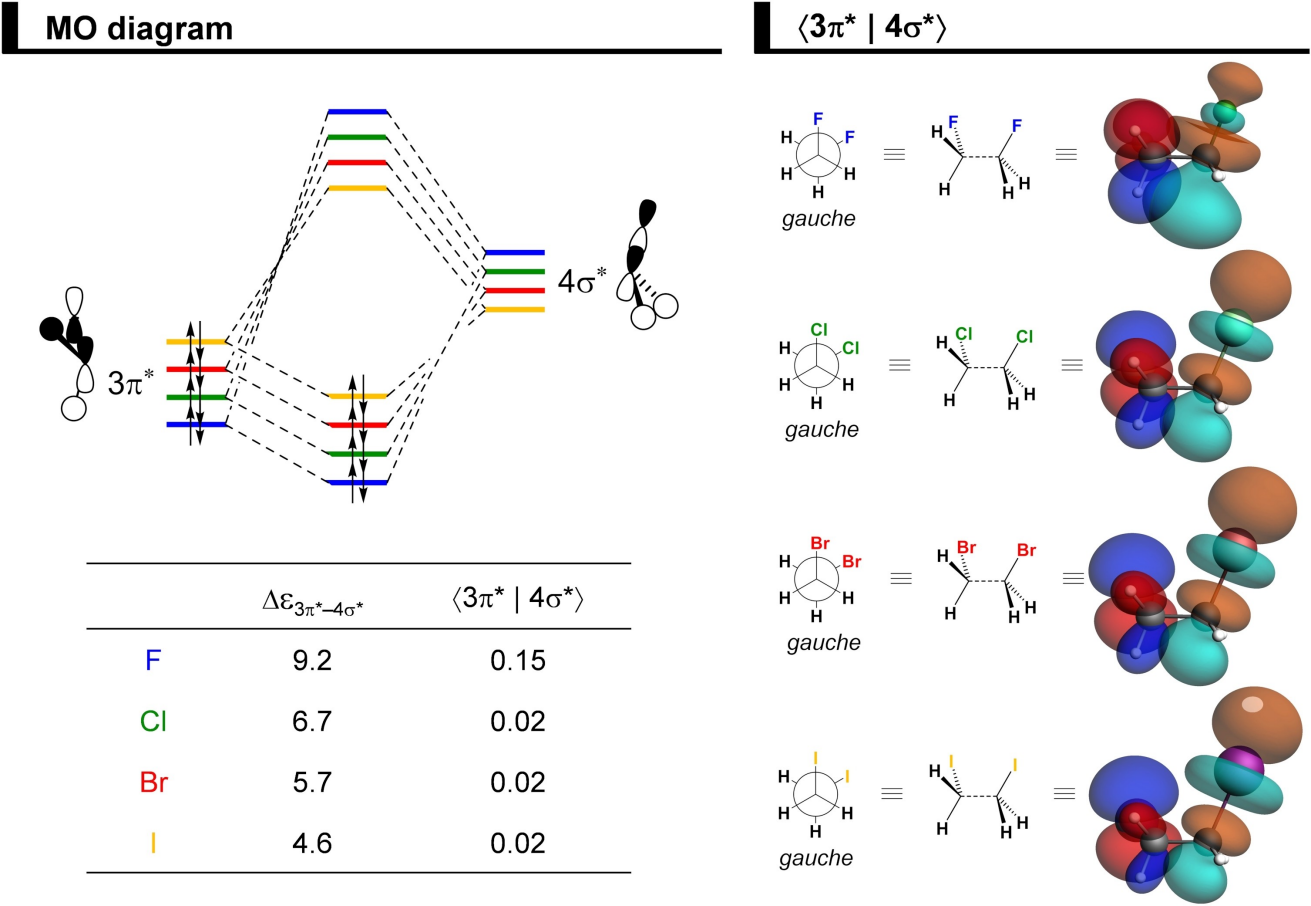
MO diagram for the donor‐acceptor interaction between the occupied 3π^*^ orbital of one fragment and the unoccupied 4σ^*^ orbital of the other fragment, along with 3π^*^ overlapping with 4σ^*^ as 3D plots (isovalue = 0.04) in the *gauche* conformers of the 1,2‐dihaloethanes. Analysis in rigid rotation in *gauche* geometry but with C−C distance set to 1.52 Å, computed at ZORA‐BP86‐D3(BJ)/QZ4P.

The hyperconjugative orbital interactions are more favorable in the *gauche* conformer, in line with the current view of the *gauche* effect.^[8]^ However, this is the case for all 1,2‐dihaloethanes analyzed herein, not only for X = F, because the orbital energy gap of the donor‐acceptor interaction favoring *gauche* (*i. e*., 3π* → 4σ*) is smaller than the one favoring *anti* (*i. e*., 2π → 4σ*; see MO diagram in Figure 2). Thus, the stabilization due to the ΔΔ*E*_oi_ results predominantly from a charge‐transfer from the occupied 3π* orbital of one fragment into the unoccupied 4σ* orbital of the other fragment (see Figure 9, or Figure S8 for the full data along the rotation of the C−C bond). This is more stabilizing for the 1,2‐difluoroethane solely because of the larger orbital overlap (⟨3π*|4σ*⟩ = 0.15 and 0.02 for X = F and I, respectively). Again, due to the difference in electronegativity in the C−F bond, the amplitude of both antibonding orbitals, 3π* and 4σ*, is larger on the less electronegative atom, that is, on carbon (see Figure 9). Thus, as the difference in electronegativity decreases on going towards heavier halogens, the orbital overlap between the two CH_2_X^.^ fragments also decreases. For the same reason, the orbital energy gap is always bigger when X = F (Δϵ = 9.2 eV for X = F and Δϵ = 4.6 eV for X = I), that is, it shows a trend opposite to that of ΔΔ*E*_oi_. This is interesting because previously the strength of hyperconjugative interactions in substituted ethanes has been typically attributed to the orbital energies alone.^[28]^ Note that the contribution from the 3σ → 4σ* is less important for the conformational preferences because of a larger orbital energy gap and the associated overlap is similar for *gauche* and *anti* (see Figures 2 and S8).

The above agrees well with the fact that the orbital interactions have the strongest preference for the *gauche* conformer of 1,2‐dihaloethanes in the case of X = F. Nevertheless, the reason that this preference of the orbital interactions for *gauche* can become decisive for X = F, is the very small difference in Pauli repulsion ΔΔ*E*_Pauli_, in that case, between *gauche* and *anti*‐conformers.

## 3 Conclusions

At variance with the current model, the *gauche* effect in 1,2‐dihaloethanes (X = F, Cl, Br, I) is not caused by hyperconjugation alone, but also by steric Pauli repulsion between substituents X, as follows from our quantum chemical analysis based on relativistic dispersion‐corrected density functional theory. The *gauche* effect refers to the phenomenon that the *gauche* conformer is energetically favored for X = F while, in all other cases (X = Cl, Br, I), the preferred conformer is *anti*. The current model ascribes the *gauche* effect to hyperconjugative orbital interactions that favor *gauche* in case of X = F. Our Kohn‐Sham molecular orbital (KS‐MO) analyses, however, reveal that such hyperconjugative orbital interactions favor the *gauche* conformation *in all cases*, not only for X = F but also for X = Cl, Br, and I. The analyses show that it is Pauli repulsion between lone‐pair‐type orbitals on the halogen substituents that constitutes the causal mechanism for the *gauche* effect. Thus, only in the case of the relatively small fluorine atoms, steric Pauli repulsion is small enough to not overrule the *gauche* preference of the hyperconjugative orbital interactions. For the larger halogens, X⋅⋅⋅X steric Pauli repulsion destabilizes the *gauche* conformer and, in this way, shifts the energetic preference from *gauche* to *anti*, despite the inverse preference of hyperconjugation.

## Conflict of interest

The authors declare no conflict of interest.

## Supporting information

As a service to our authors and readers, this journal provides supporting information supplied by the authors. Such materials are peer reviewed and may be re‐organized for online delivery, but are not copy‐edited or typeset. Technical support issues arising from supporting information (other than missing files) should be addressed to the authors.

Supplementary
