# Supplementary material for: The Gauche Effect in XCH2CH2X Revisited
Source: Chemphyschem. 2021 Feb 25;22(7):641–8. doi: 10.1002/cphc.202100090 (PMC8048458; doi:10.1002/cphc.202100090)
Supplement: Supplementary file 1 — Supplementary [file CPHC-22-641-s001.pdf]

ChemPhysChem

Supporting Information

The *Gauche* Effect in XCH_2CH_2X Revisited

Daniela Rodrigues Silva[+], Lucas de Azevedo Santos[+], Trevor A. Hamlin, Célia Fonseca Guerra,* Matheus P. Freitas,* and F. Matthias Bickelhaupt*

Table of contents

Computational Details

Figure S1. MO diagram for the formation of $H_2C^{\bullet}$–X (left side) and H_3C–X (right side) from $X^{\bullet}$ (= $F^{\bullet}$, $Cl^{\bullet}$, $Br^{\bullet}$, $I^{\bullet}$) interacting with $H_2C^{\bullet\bullet}$ and $H_3C^{\bullet}$, respectively.

Figure S2. Rotational energy profile and C–C bond length variation as a function of the $\varphi_{X–C–C–X}$ dihedral angle of the 1,2-dihaloethanes $XH_2C–CH_2X$ (X = F, Cl, Br, I), computed at ZORA-BP86-D3(BJ)/QZ4P.

Figure S3. EDA of the interaction between two open-shell $CH_2X^{\bullet}$ fragments in 1,2-dihaloethanes $XH_2C–CH_2X$ as a function of the C–C distance (X = F, Cl, Br, I), computed at ZORA-BP86-D3(BJ)/QZ4P.

Figure S4. Derivative of the EDA terms with respect to $r_{C–C}$ of the interaction between two open-shell $CH_2X^{\bullet}$ fragments in 1,2-dihaloethanes $XH_2C–CH_2X$ as a function of the C–C separation (X = F, Cl, Br, I). Computed at ZORA-BP86-D3(BJ)/QZ4P. Since the ΔV_{elstat} and ΔE_{oi} curves are almost superposing each other in some cases, the orbital interaction curves are slightly transparent for clarity.

Figure S5. Activation strain (ASA) and energy decomposition analyses (EDA) as a function of the $\varphi_{X–C–C–X}$ dihedral angle of the 1,2-dihaloethanes $XH_2C–CH_2X$ (X = F, Cl, Br, I). a) ASA and b) EDA for fully relaxed rotation, c) EDA and d) key occupied–occupied orbital overlaps for rigid rotation in *gauche* geometry but with C–C distance set to 1.52 Å. Computed at ZORA-BP86-D3(BJ)/QZ4P.

Figure S6. EDA as a function of the $\varphi_{X–C–C–X}$ dihedral angle of the 1,2-dihaloethanes $XH_2C–CH_2X$ (X = F, Cl, Br, I) for rigid rotation in frozen *gauche* (top) and *anti* (bottom) geometries, computed at ZORA-BP86-D3(BJ)/QZ4P.

Figure S7. Electrostatic interaction, Pauli repulsion, and orbital interaction energy terms as a function of the $\varphi_{X–C–C–X}$ dihedral angle of the 1,2-dihaloethanes $XH_2C–CH_2X$ (X = F, Cl, Br, I). Full lines: EDA for fully relaxed rotation; doted lines: EDA for rigid rotation in frozen *gauche* geometry; dashed lines: EDA for rigid rotation in frozen *syn* geometry. Computed at ZORA-BP86-D3(BJ)/QZ4P.

Table S1. EDA terms (in kcal mol^{-1}) of the energy minimum stationary points relative to the *syn* conformer in rigid rotation around the C–C bond in *gauche* geometry but with C–C distance set to 1.52 Å.

Figure S8. Orbital interactions along with the gross population of the $4\sigma^*$ orbital and the main occupied–unoccupied orbital overlaps as a function of the $\varphi_{X–C–C–X}$ dihedral angle of the 1,2-dihaloethanes $XH_2C–CH_2X$ (X = F, Cl, Br, I). Analysis in rigid rotation in *gauche* geometry with C–C distance set to 1.52 Å. Computed at ZORA-BP86-D3(BJ)/QZ4P.

Table S2. Cartesian coordinates (Å), energies (kcal mol^{-1}), and the number of imaginary vibrational frequencies (N_{imag}) of stationary points of 1,2-dihaloethanes, computed at ZORA-BP86-D3(BJ)/QZ4P.

Computational Details

All calculations were performed using the Amsterdam Density Functional (ADF) program 2017.103.[1] Geometry optimization and vibrational analysis of the energy minimum conformations and rotational barrier analysis were carried out using the dispersion-corrected BP86[2]-D3(BJ)[3] functional in conjunction with the quadruple-ζ quality augmented with polarization functions (two 2*p* and two 3*d* sets on H, two 3*d* and two 4*f* sets on C, F; three 3*d* and two 4*f* sets on Cl, two 4*d* and three 4*f* sets on Br, one 5*d* and three 4*f* sets on I) QZ4P basis set.[4] The zeroth-order regular approximation (ZORA)[5] was used to account for scalar relativistic effects. This level is referred to as ZORA-BP86-D3(BJ)/QZ4P. The activation strain and energy decomposition analyses were performed using the PyFrag program at the same level of theory.[6]

---

[1] a) G. te Velde, F. M. Bickelhaupt, E. J. Baerends, C. Fonseca Guerra, S. J. A. van Gisbergen, J. G. Snijders, T. Ziegler, *J. Comput. Chem.* **2001**, *22*, 931; b) C. Fonseca Guerra, J. G. Snijders, G. te Velde, E. J. Baerends, *Theor. Chem. Acc.* **1998**, *99*, 391; c) ADF2017.103, SCM Theoretical Chemistry, Vrije Universiteit: Amsterdam (Netherlands), 2010. http:// www.scm.com.

[2] a) A. D. Becke, *Phys. Rev. A* **1988**, *38*, 3098; b) J. P. Perdew, *Phys. Rev. B: Condens. Matter Mater. Phys.* **1986**, *33*, 8822.

[3] a) S. Grimme, J. Antony, S. Ehrlich, H. Krieg, *J. Chem. Phys.* **2010**, *132*, 154104; b) S. Grimme, S. Ehrlich, L. Goerigk, *J. Comput. Chem.* **2011**, *32*, 1456.

[4] E. van Lenthe, E. J. Baerends, *J. Comput. Chem.* **2003**, *24*, 1142.

[5] a) E. van Lenthe, E. J. Baerends, J. G. Snijders, *J. Chem. Phys.* **1993**, *99*, 4597; b) E. van Lenthe, E. J. Baerends, J. G. Snijders, *J. Chem. Phys.* **1994**, *101*, 9783.

[6] a) W. -J. van Zeist, C. Fonseca Guerra, F. M. Bickelhaupt, *J. Comp. Chem.* **2008**, *29*, 312; b) X. Sun, T. M. Soini, J. Poater, T. A. Hamlin, F. M. Bickelhaupt, *J. Comp. Chem.* **2019**, *40*, 2227.

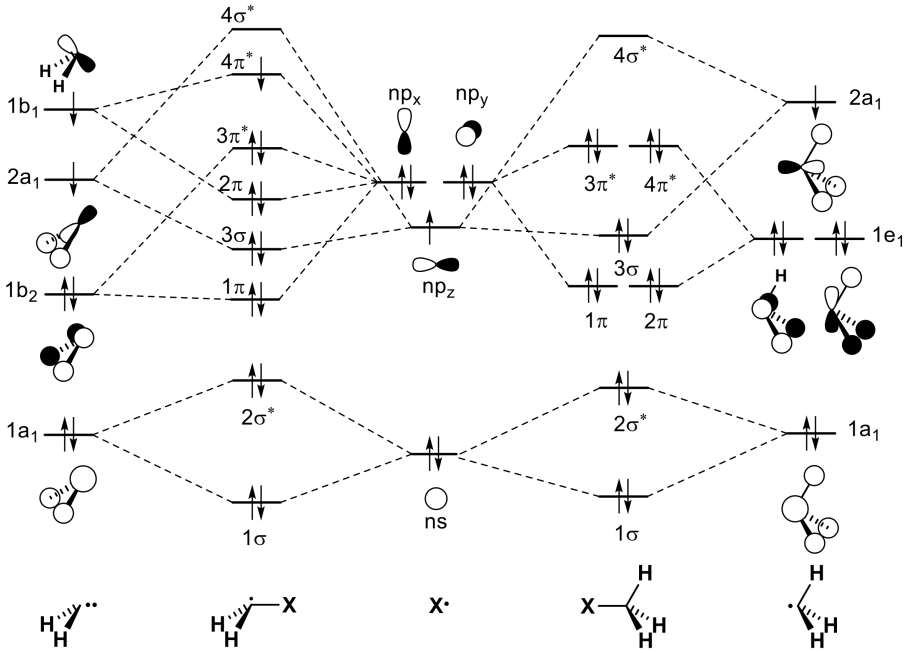

Figure S1. MO diagram for the formation of $H_2C^{\bullet}$–X (left side) and H_3C–X (right side) from $X^{\bullet}$ (= $F^{\bullet}$, $Cl^{\bullet}$, $Br^{\bullet}$, $I^{\bullet}$) interacting with $H_2C^{\bullet\bullet}$ and $H_3C^{\bullet}$, respectively.

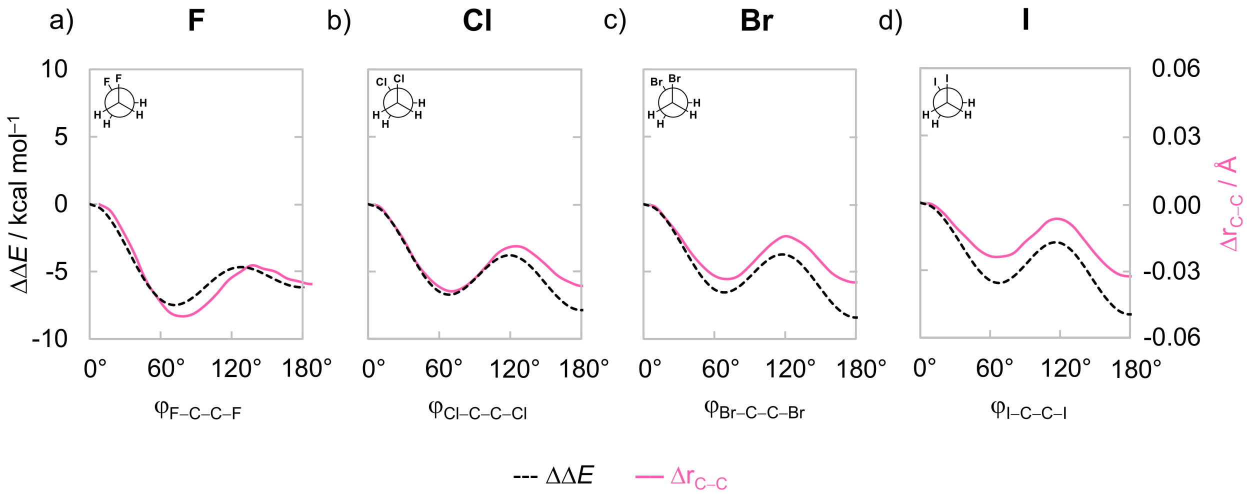

Figure S2. Rotational energy profile and C–C bond length variation as a function of the $\varphi_{X\text{–}C\text{–}C\text{–}X}$ dihedral angle of the 1,2-dihaloethanes XH_2C–CH_2X (X = F, Cl, Br, I), computed at ZORA-BP86-D3(BJ)/QZ4P.

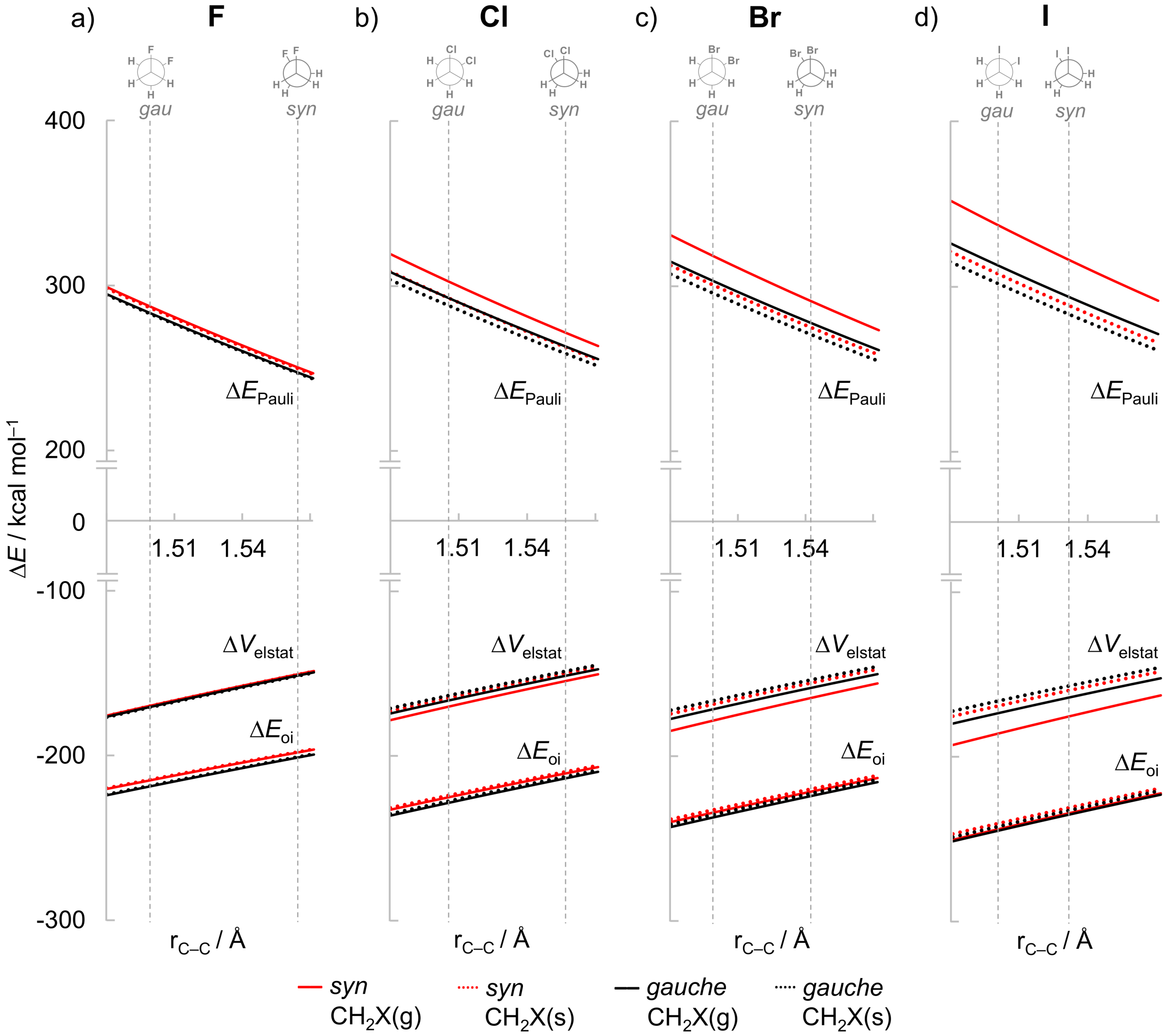

Figure S3. EDA of the interaction between two open-shell $CH_2X^{\bullet}$ fragments in 1,2-dihaloethanes $XH_2C–CH_2X$ as a function of the C–C distance (X = F, Cl, Br, I), computed at ZORA-BP86-D3(BJ)/QZ4P.

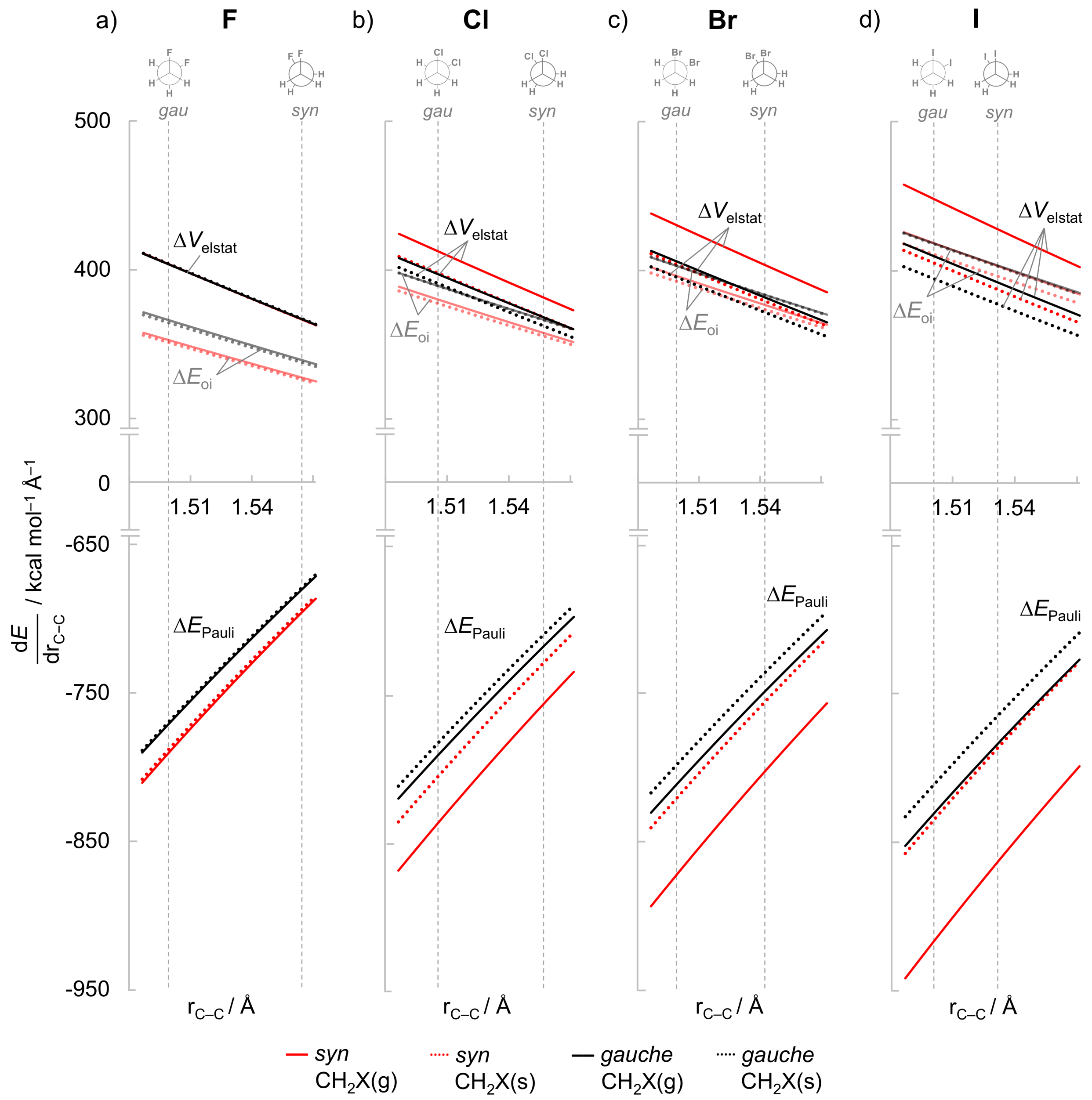

Figure S4. Derivative of the EDA terms with respect to $r_{C–C}$ of the interaction between two open-shell $CH_2X^{\bullet}$ fragments in 1,2-dihaloethanes $XH_2C–CH_2X$ as a function of the C–C separation (X = F, Cl, Br, **I**). Computed at ZORA-BP86-D3(BJ)/QZ4P. Since the ΔV_{elstat} and ΔE_{oi} curves are almost superposing each other in some cases, the orbital interaction curves are slightly transparent for clarity.

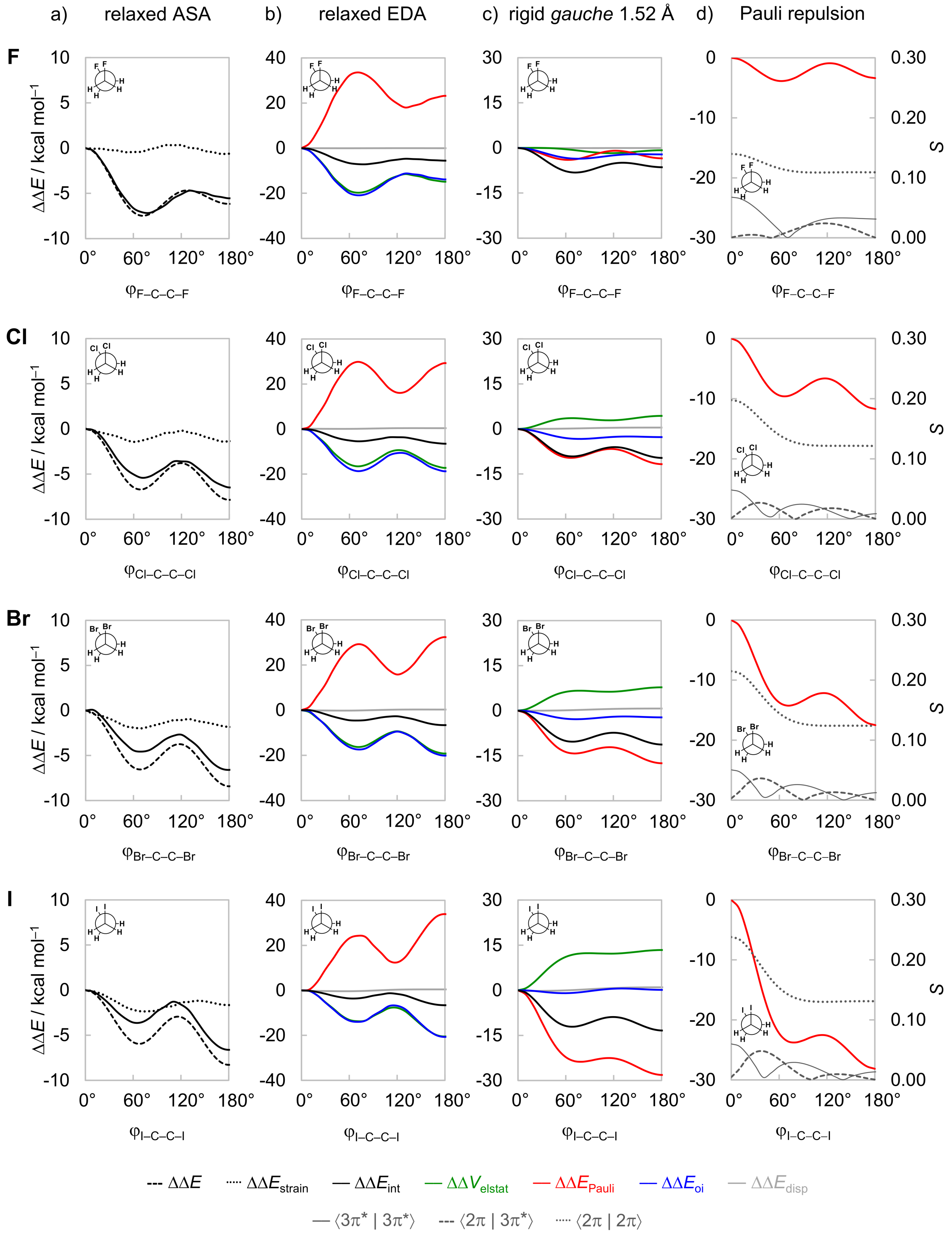

Figure S5. Activation strain (ASA) and energy decomposition analyses (EDA) as a function of the $\varphi_{X–C–C–X}$ dihedral angle of the 1,2-dihaloethanes $XH_2C–CH_2X$ (X = F, Cl, Br, I). a) ASA and b) EDA for fully relaxed rotation, c) EDA and d) key occupied–occupied orbital overlaps for rigid rotation in *gauche* geometry but with C–C distance set to 1.52 Å. Computed at ZORA-BP86-D3(BJ)/QZ4P.

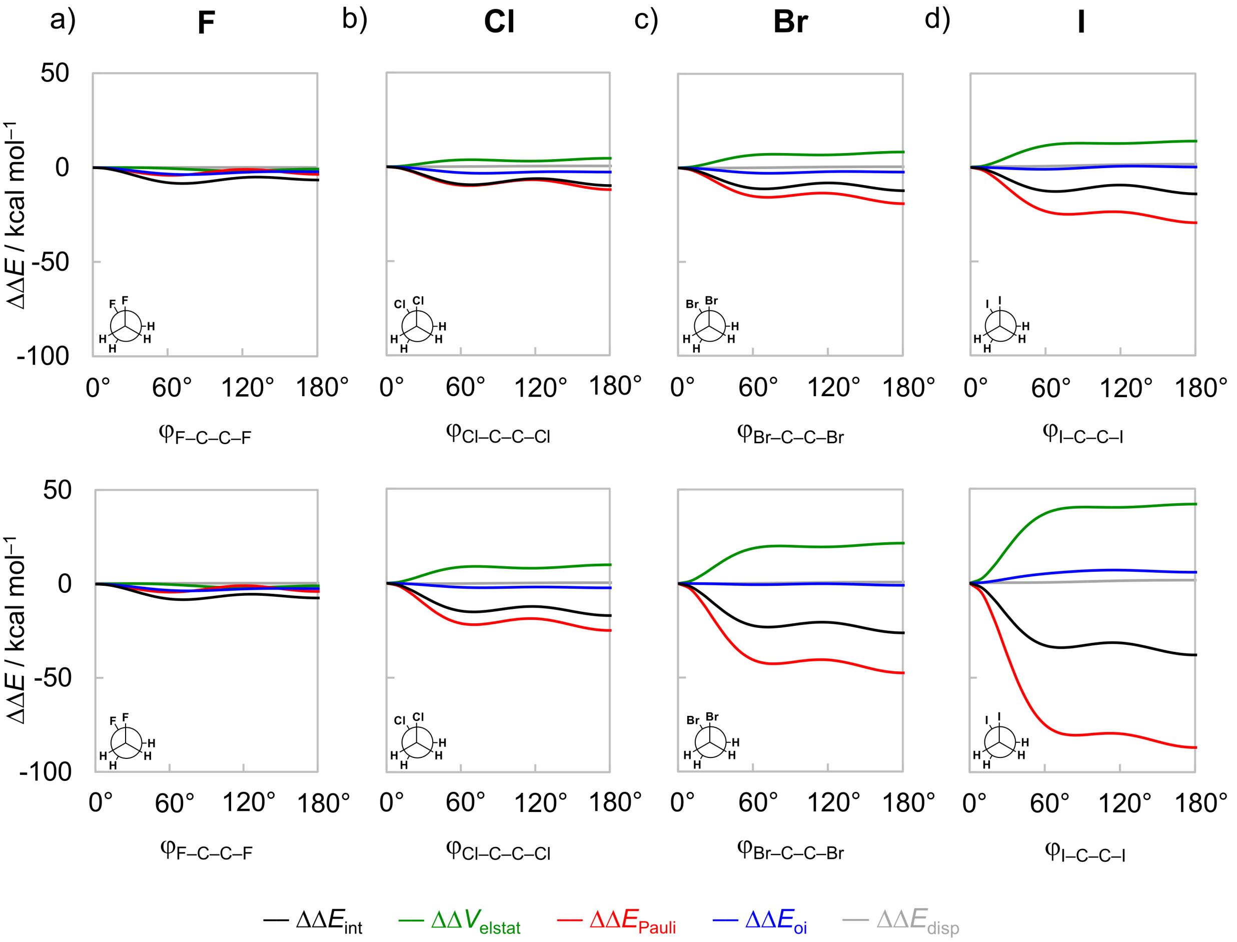

Figure S6. EDA as a function of the $\varphi_{X-C-C-X}$ dihedral angle of the 1,2-dihaloethanes XH_2C-CH_2X (X = F, Cl, Br, I) for rigid rotation in frozen *gauche* (top) and *anti* (bottom) geometries, computed at ZORA-BP86-D3(BJ)/QZ4P.

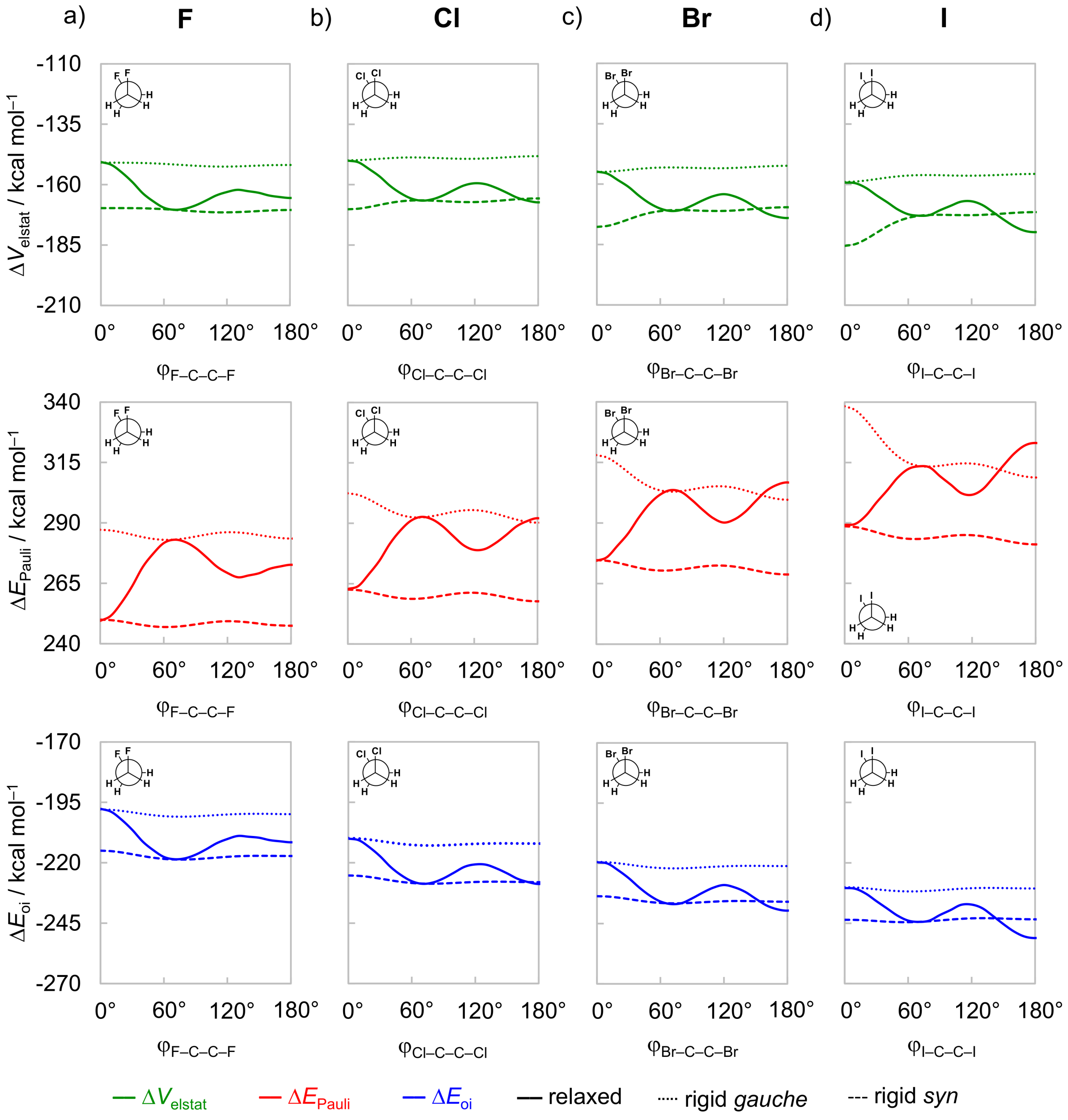

Figure S7. Electrostatic interaction, Pauli repulsion, and orbital interaction energy terms as a function of the $\varphi_{X\text{–}C\text{–}C\text{–}X}$ dihedral angle of the 1,2-dihaloethanes $XH_2C–CH_2X$ (X = F, Cl, Br, I). Full lines: EDA for fully relaxed rotation; doted lines: EDA for rigid rotation in frozen *gauche* geometry; dashed lines: EDA for rigid rotation in frozen *syn* geometry. Computed at ZORA-BP86-D3(BJ)/QZ4P.

Table S1. EDA terms (in kcal mol−1) of the energy minimum stationary points relative to the *syn* conformer in rigid rotation around the C–C bond in *gauche* geometry but with C–C distance set to 1.52 Å.[a]

X	$\Delta\Delta E_{int}$		$\Delta\Delta V_{elstat}$		$\Delta\Delta E_{Pauli}$		$\Delta\Delta E_{oi}$		$\Delta\Delta E_{disp}$	
	gau	anti	gau	anti	gau	anti	gau	anti	gau	anti
**F**	–8.0	–6.3	–0.7	–0.8	–3.8	–3.4	–3.5	–2.1	0.0	0.0
**Cl**	–9.1	–9.6	3.6	4.3	–9.6	–11.7	–3.3	–2.7	0.1	0.5
**Br**	–10.3	–11.3	6.6	7.8	–10.3	–11.3	–2.8	–2.2	0.2	0.7
**I**	–12.1	–13.4	11.9	13.4	–23.5	–28.1	–0.9	0.1	0.3	1.2

[a] Compute at ZORA-BP86-D3(BJ)/QZ4P.

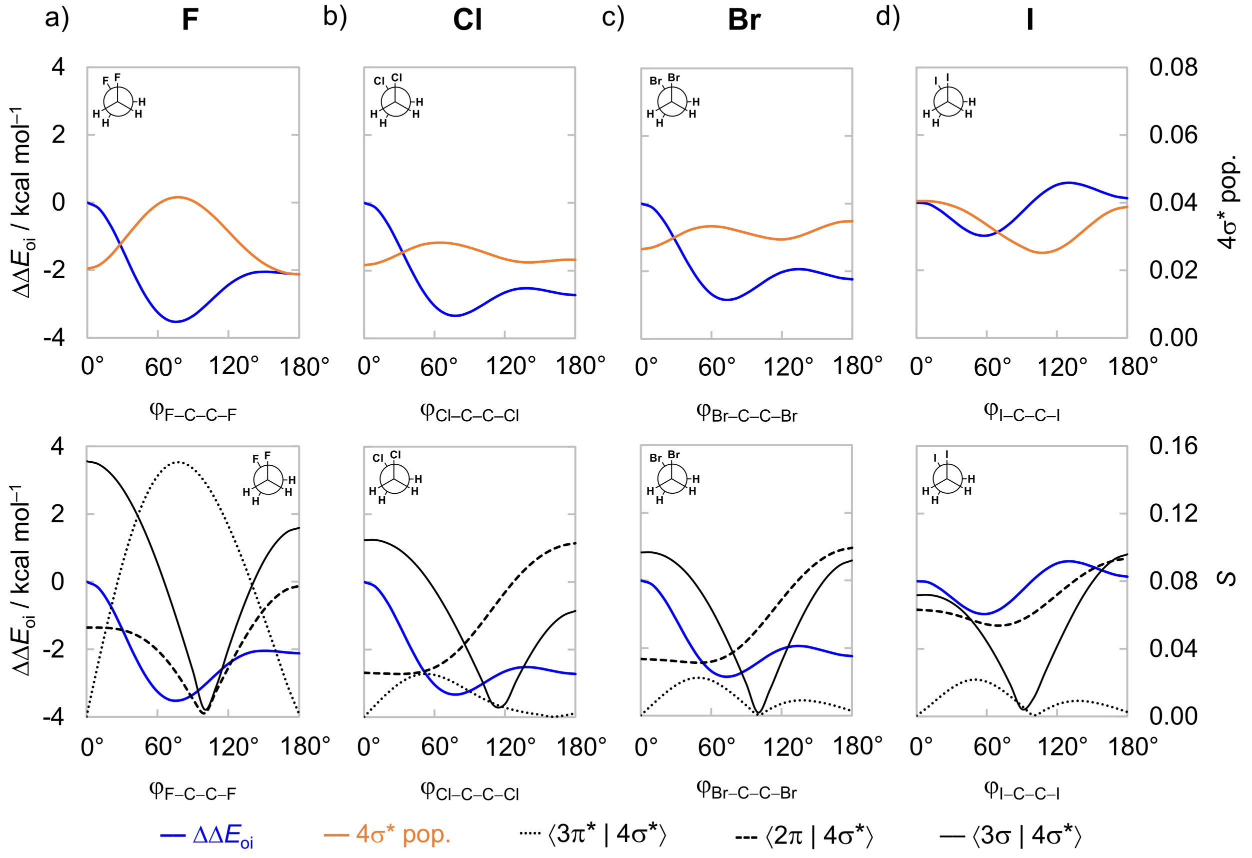

Figure S8. Orbital interactions along with the gross population of the 4σ* orbital and the main occupied–unoccupied orbital overlaps as a function of the $\varphi_{X-C-C-X}$ dihedral angle of the 1,2-dihaloethanes XH_2C-CH_2X (X = F, Cl, Br, I). Analysis in rigid rotation in *gauche* geometry with C–C distance set to 1.52 Å. Computed at ZORA-BP86-D3(BJ)/QZ4P.

Table S2. Cartesian coordinates (Å), energies (kcal mol^{-1}), and the number of imaginary vibrational frequencies (N_{imag}) of stationary points of 1,2-dihaloethanes, computed at ZORA-BP86-D3(BJ)/QZ4P.

Syn **1,2-difluoroethane**

E = –927.4

N_{imag} = 1, ν = –215.2658i cm^{-1}

C	0.00000074	-0.77762570	0.07525757
C	-0.00000074	0.77762570	0.07525757
H	0.89491724	-1.16732643	0.57926708
H	-0.89491490	-1.16732810	0.57926732
H	-0.89491724	1.16732643	0.57926710
H	0.89491490	1.16732809	0.57926733
F	0.00000099	-1.27638234	-1.22458875
F	-0.00000099	1.27638234	-1.22458875

Gauche **1,2-difluoroethane**

E = –934.9

N_{imag} = 0

C	0.32161802	0.68072665	0.03290025
H	1.41167724	0.59913582	-0.08134650
H	0.08565883	1.20093536	0.97399384
C	-0.32161802	-0.68072665	0.03290025
H	-1.41167724	-0.59913582	-0.08134650
H	-0.08565883	-1.20093536	0.97399384
F	0.16171168	-1.46275699	-1.02473867
F	-0.16171168	1.46275699	-1.02473867

Anticlinal **1,2-difluoroethane**

E = –932.1

N_{imag} = 1, ν = –118.9539i cm^{-1}

C	-0.40802527	-0.64577750	-0.32158344
C	0.40805434	0.64578023	-0.32162585
H	-0.68481999	-0.97330352	0.68875597
H	-1.31422138	-0.49809044	-0.92436459
H	0.68494061	0.97331482	0.68868573
H	1.31419581	0.49808814	-0.92448788
F	0.33895153	-1.68592872	-0.89996460
F	-0.33897493	1.68592652	-0.89994819

***Anti* 1,2-difluoroethane**

E = –933.6

N_{imag} = 0

C	0.55333841	0.52120709	-0.00000000
H	0.50382924	1.15154326	0.89793956
H	0.50382924	1.15154326	-0.89793956
C	-0.55333841	-0.52120709	-0.00000000
H	-0.50382924	-1.15154326	-0.89793956
H	-0.50382924	-1.15154326	0.89793956
F	1.78599548	-0.14957726	0.00000000
F	-1.78599548	0.14957726	0.00000000

***Syn* 1,2-dichloroethane**

E = –849.6

N_{imag} = 1, ν = –189.0550i cm^{-1}

C	-0.00001604	-0.77525086	0.29544878
C	0.00001604	0.77525086	0.29544876
H	0.88893482	-1.14536558	0.81335082
H	-0.88898252	-1.14532878	0.81335034
H	-0.88893481	1.14536558	0.81335083
H	0.88898251	1.14532878	0.81335035
Cl	-0.00003224	-1.57578524	-1.30856958
Cl	0.00003224	1.57578524	-1.30856957

***Gauche* 1,2-dichloroethane**

E = –856.5

N_{imag} = 0

C	0.29990324	0.69358194	0.06539914
H	1.39126015	0.66024554	-0.00284582
H	0.00668527	1.21593809	0.98389572
C	-0.29990324	-0.69358194	0.06539914
H	-1.39126015	-0.66024554	-0.00284582
H	-0.00668527	-1.21593809	0.98389572
Cl	0.28381509	-1.69895780	-1.30874193
Cl	-0.28381509	1.69895780	-1.30874193

***Anticlinal* 1,2-dichloroethane**

E = –853.6

N_{imag} = 1, ν = –106.0801i cm^{-1}

C	-0.49301277	-0.58553566	-0.23443226

C	0.49301212	0.58553570	-0.23443158
H	-0.72723260	-0.91016083	0.78342185
H	-1.41918903	-0.32473695	-0.75139728
H	0.72723075	0.91016091	0.78342279
H	1.41918899	0.32473702	-0.75139554
Cl	0.18087257	-2.03123590	-1.08077468
Cl	-0.18087229	2.03123588	-1.08077490

Anti **1,2-dichloroethane**

E = –857.7

$\boldsymbol{N_{imag}}$ = 0

C	0.55114025	0.51922017	0.00000001
H	0.51374108	1.14831221	0.89361310
H	0.51374108	1.14831221	-0.89361304
C	-0.55114025	-0.51922017	0.00000001
H	-0.51374108	-1.14831221	-0.89361304
H	-0.51374108	-1.14831221	0.89361310
Cl	2.14835262	-0.32506156	-0.00000000
Cl	-2.14835262	0.32506156	-0.00000000

Syn **1,2-dibromoethane**

E = –823.4

$\boldsymbol{N_{imag}}$ = 1, ν = –177.7019i cm^{-1}

C	-0.00000294	-0.76938848	0.45049953
C	0.00000294	0.76938853	0.45049956
H	0.89082521	-1.14863949	0.95530467
H	-0.89083407	-1.14863269	0.95530452
H	-0.89082521	1.14863956	0.95530468
H	0.89083408	1.14863275	0.95530453
Br	-0.00000635	-1.69341836	-1.28281639
Br	0.00000635	1.69341835	-1.28281638

Gauche **1,2-dibromoethane**

E = –830.1

$\boldsymbol{N_{imag}}$ = 0

C	0.29819271	0.69315392	0.05774382
H	1.38557309	0.68589586	-0.04685831
H	0.01599997	1.22631228	0.97223779
C	-0.28944360	-0.69315392	0.07321150
H	-1.38082109	-0.68589586	0.02595835

H	0.04046743	-1.22631228	0.97159377
Br	0.31224911	-1.79664350	-1.43895183
Br	-0.38221763	1.79664350	-1.42067216

***Anticlinal* 1,2-dibromoethane**

E = –827.3

N_{imag} = 1, ν = –86.9701i cm^{-1}

C	-0.52987018	-0.54730098	-0.20457111
C	0.52987769	0.54730095	-0.20457612
H	-0.76282891	-0.88499867	0.80833287
H	-1.44753386	-0.24383345	-0.71010654
H	0.76284594	0.88499863	0.80832569
H	1.44753660	0.24383341	-0.71012022
Br	0.08069064	-2.16258407	-1.16006387
Br	-0.08069204	2.16258408	-1.16006314

***Anti* 1,2-dibromoethane**

E = –832.1

N_{imag} = 0

C	0.54239947	0.52062565	0.00000000
H	0.53088295	1.14295745	0.89698219
H	0.53088295	1.14295745	-0.89698219
C	-0.54239947	-0.52062565	0.00000000
H	-0.53088295	-1.14295745	-0.89698219
H	-0.53088295	-1.14295745	0.89698219
Br	2.29263568	-0.40674418	-0.00000000
Br	-2.29263568	0.40674418	-0.00000000

***Syn* 1,2-diiodoethane**

E = –800.8

N_{imag} = 1, ν = –167.2958i cm^{-1}

C	-0.00002313	-0.76561734	0.60838336
C	0.00002313	0.76561737	0.60838338
H	0.89039680	-1.14862387	1.11019360
H	-0.89047059	-1.14856975	1.11018639
H	-0.89039681	1.14862389	1.11019359
H	0.89047060	1.14856978	1.11018641
I	-0.00005148	-1.85296775	-1.27398782
I	0.00005148	1.85296775	-1.27398781

***Gauche* 1,2-diiodoethane**

E = –807.0

$\boldsymbol{N_{imag}}$ = 0

C	0.28376254	0.69773287	0.06470180
H	1.37371361	0.71320003	-0.00405361
H	-0.03786848	1.23488915	0.96391767
C	-0.28376254	-0.69773287	0.06470180
H	-1.37371361	-0.71320003	-0.00405361
H	0.03786848	-1.23488915	0.96391767
I	0.42205298	-1.92611551	-1.57921910
I	-0.42205298	1.92611551	-1.57921910

***Anticlinal* 1,2-diiodoethane**

E = –803.9

$\boldsymbol{N_{imag}}$ = 1, ν = –71.3354i cm^{-1}

C	-0.54623363	-0.53057214	-0.11960789
C	0.54625136	0.53057191	-0.11961838
H	-0.77891115	-0.87053024	0.89262186
H	-1.46411774	-0.21363484	-0.61492535
H	0.77894816	0.87053002	0.89260691
H	1.46412588	0.21363452	-0.61495357
I	0.04724048	-2.33116094	-1.18972125
I	-0.04724252	2.33116096	-1.18972029

***Anti* 1,2-diiodoethane**

E = –809.5

$\boldsymbol{N_{imag}}$ = 0

C	0.54162651	0.51722443	-0.00000000
H	0.54745334	1.13908207	0.89671659
H	0.54745334	1.13908207	-0.89671659
C	-0.54162651	-0.51722443	-0.00000000
H	-0.54745334	-1.13908207	-0.89671659
H	-0.54745334	-1.13908207	0.89671659
I	2.48052788	-0.50543189	0.00000000
I	-2.48052788	0.50543189	0.00000000
